# Experience in prenatal ultrasound diagnosis of fetal microtia and associated abnormalities

**DOI:** 10.3389/fmed.2023.1119191

**Published:** 2023-07-11

**Authors:** Jing Qiu, Yanhui Ru, Yang Gao, Jie Shen

**Affiliations:** ^1^Graduate School, Tianjin Medical University, Tianjin, China; ^2^Department of Nuclear Medicine, The First Central Clinical School, Tianjin Medical University, Tianjin, China; ^3^Department of Ultrasound, Shandong Provincial Maternal and Child Health Care Hospital, Jinan, China

**Keywords:** microtia, ultrasonic soft index, prenatal ultrasound, associated anomalies, genetic abnormalities

## Abstract

**Objective:**

Prenatal ultrasound features, associated anomalies and genetic abnormalities of microtia cases were analyzed to explore the feasibility and value of prenatal ultrasound for the diagnosis of microtia.

**Methods:**

The ultrasonographic features, associated anomalies, chromosome examination results and follow-up results of 81 fetuses with congenital microtia were analyzed retrospectively.

**Results:**

Among the 81 fetuses with microtia diagnosed after birth, 2 cases were missed diagnosis on prenatal ultrasound, and 1 case was diagnosed as unilateral microtia by prenatal ultrasound but was found to be bilateral microtia after birth. Microtia was accompanied by an accessory auricle in 4 cases (4.94%) and low-set ears in 7 cases (8.64%). 22 cases (27.16%) were complicated with other structural anomalies, including 11 cases (13.58%) of cardiac anomalies, 7 cases (8.64%) of ultrasonographic soft marker anomalies, 6 cases (7.41%) of facial anomalies, 6 cases (7.41%) of nervous system anomalies, 3 cases (3.70%) of urogenital system anomalies, 3 cases (3.70%) of digestive tract anomalies and 2 cases (2.47%) of limb anomalies. Chromosome karyotype analysis and gene detection were performed in 44 cases. Trisomy 18, trisomy 13, trisomy 21, pericentric inversion of chromosome 9, partial loss of heterozygosity on chromosome 14, 22q11 microdeletion and a normal karyotype were found in 2 cases, 2 cases, 3 cases, 1 case, 1 case, 1 case, and 34 cases, respectively.

**Conclusion:**

In summary, microtia is often accompanied by congenital defects of other organs and structures, especially the heart and face, and prenatal ultrasound diagnosis of microtia and associated anomalies is of important clinical significance.

## 1. Introduction

Congenital microtia is a common congenital malformation presenting with external birth defects, which are also often accompanied by atresia and stricture of the external acoustic meatus and middle ear deformity, leading to conductive deafness and affecting the development of hearing and language. An external auricle length less than twice the standard deviation of the mean fetal auricle length at the same gestational age indicates microtia (1, 2).

Fetal ear examination is not a routine item in prenatal ultrasound screening, but microtia and associated anomalies can be detected by prenatal ultrasound, thereby guiding patients to receive targeted screening and providing a basis for prognostic evaluations of fetuses with microtia.

In this study, fetuses with microtia in our study site from July 2017 to July 2022 were retrospectively analyzed, and ultrasound features, common associated anomalies, and genetic features are summarized.

## 2. Materials and methods

### 2.1. Patients

Fetuses diagnosed with microtia by postnatal diagnosis in our hospital from July 2017 to July 2022 were selected.

The inclusion criteria were as follows: (1) fetuses whose mothers had a definite gestational age (determined by the last menstrual period and ultrasound in the first trimester), (2) those diagnosed with microtia that was confirmed after birth or induced labor, and (3) those with good-quality images that could be retrospectively analyzed. 81 fetuses meeting the diagnostic criteria for microtia by prenatal ultrasound were included in the study. Two cases were missed by prenatal ultrasound. One case was one of monochorionic diamniotic twin pregnancies, and the other 80 cases were singleton pregnancies. The median age of the pregnant women was 30 years (19 ~ 39 years), and the median gestational age at diagnosis was 24^+1^ weeks (15^+1^ ~ 33^+3^ weeks).

### 2.2. Image acquisition

Ultrasonography was performed using a Voluson E10 ultrasonography machine (GE Healthcare, Zipf, Austria), with a RAB2-5 (2.5 ~ 5 MHz) transabdominal volume convex probe.

**Figure 1:**
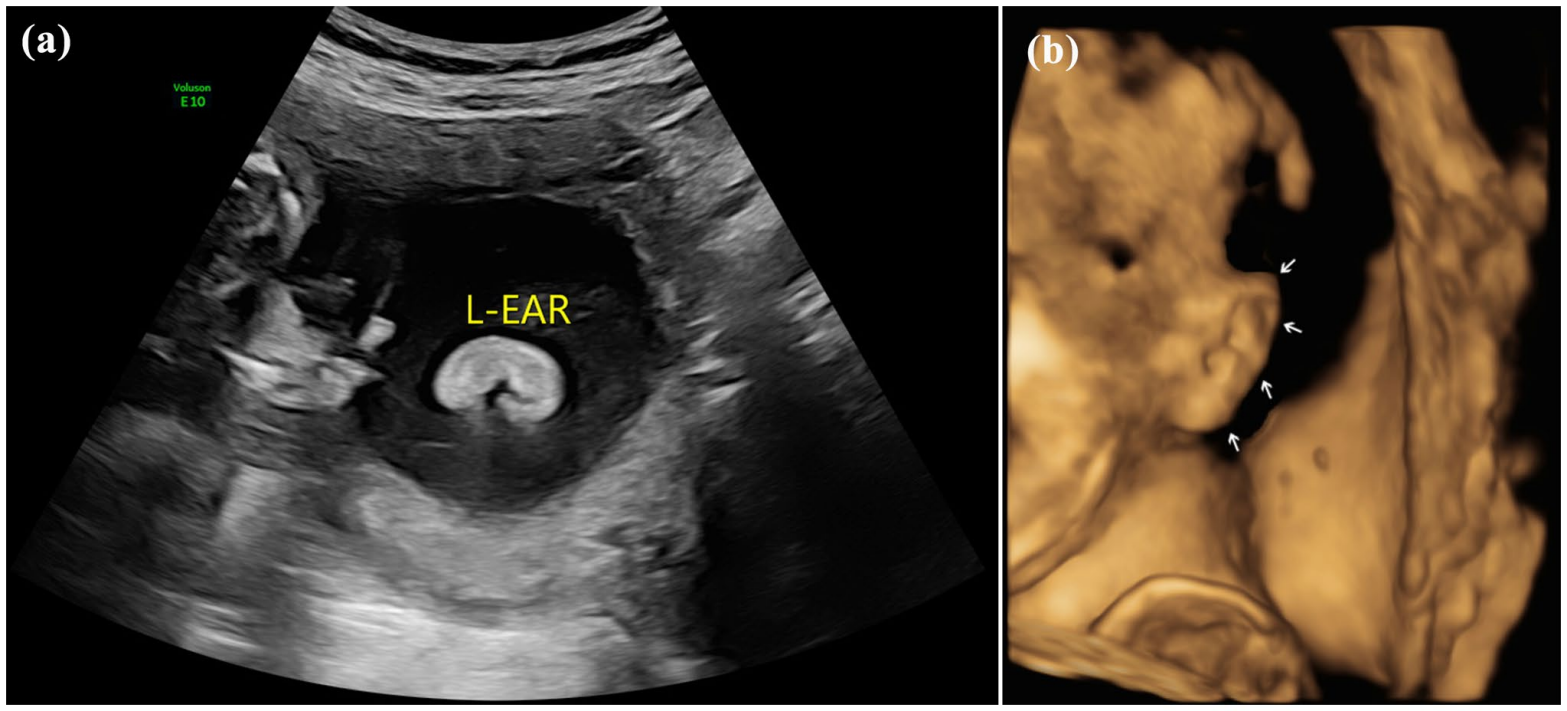
Images of normal fetal ear: **(A)** 2D sonography and **(B)** 3D sonography.

Pregnant women were placed in the supine position under obstetric conditions. The structural development of the fetuses was systematically screened by ultrasound, and biological diameters were measured. In our study site, fetal auricles were generally observed on the parasagittal plane of the temporal bone, and the probe was deflected left and right on the sagittal plane of the fetal brain or face to display the bilateral auricles. Multiplane scans (including the coronal plane and cervical posterior transverse oblique plane) and 3D imaging were further performed in cases with suspected auricle abnormality (Figure 1).

### 2.3. Methods

The position, symmetry, size and shape of the bilateral auricles were observed. If the above structures of the fetus failed to be observed due to fetal position, the pregnant woman was instructed to change her position or wait for the fetus to turn to an observable position. In cases of suspected microtia, the length of the auricle was measured 3 times and averaged. The principle of minimum energy for prenatal ultrasound diagnosis was followed.

The cases meeting the inclusion criteria were screened, as summarized in Table 1 and their ultrasound images were retrospectively analyzed. The following variables were analyzed and summarized: maternal age, gestational age at diagnosis, ultrasound features, associated anomalies, and genetic features.

**Table 1:** Types of 81 fetuses with congenital microtia.

Type of microtia	*n*	Complicated with other structural anomalies (*n*)	Genetic abnormalities (*n*)
I	1	0	0
II	17	6	1
III	62	14	9
IV	3	2	0

Bilateral microtia was found in 2 cases and unilateral microtia in 79 cases. Forty four cases performed chromosome karyotype analysis and gene detection.

## 3. Results

**Figure 2:**
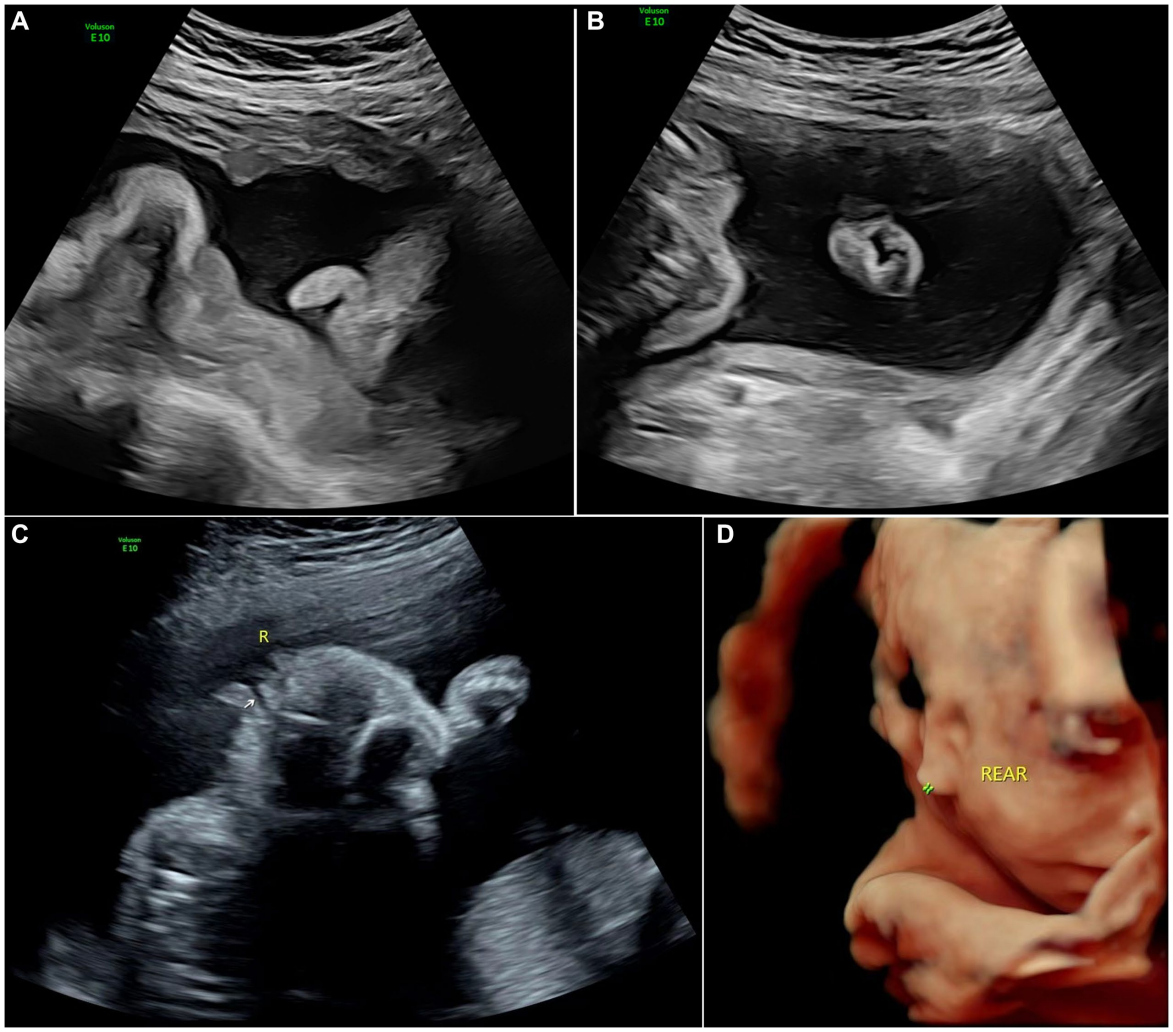
Images of type II of microtia: **(A)** 2D sonography of microtia; **(B)** 2D sonography of contralateral ear(normal); **(C)** 2D sonography of unilateral microtia type II with normal external acoustic meatus; and **(D)** 3D sonography of Unilateral microtia type II.

**Figure 3:**
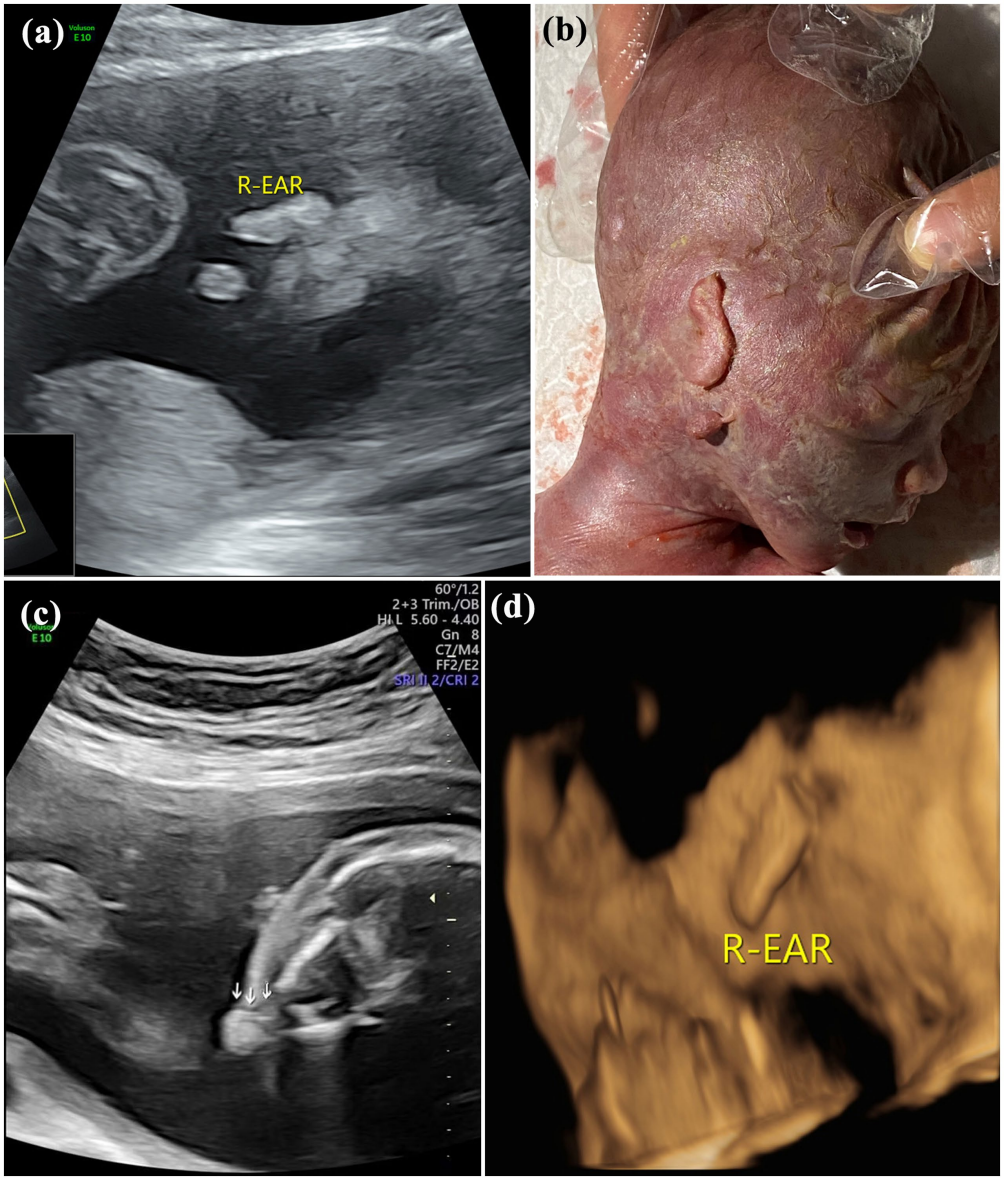
Images of type III of microtia: **(A)** 2D sonography and **(B)** photo (induced labor) of microtia type III accompanied by an accessory auricle; **(C)** 2D sonography of unilateral microtia type III accompanied by atresia of external acoustic meatus; and **(D)** 3D sonography of Unilateral microtia type III.

As indicated in research literature on the standard for the normal diameter of the fetal auricle, an external auricle length less than twice the standard deviation of the mean fetal auricle length at the same gestational age indicates microtia (1). In this study, all 81 cases met the diagnostic criteria for microtia and were accompanied by varying degrees of morphological abnormalities. Microtia can be morphologically classified into 4 types from mild to severe: type I: mild deformity and a slightly small auricle with a clear structure; type II: moderate deformity and a small auricle with a partially preserved structure (Figure 2); type III: severe deformity with only partial auricular cartilage and earlobe preserved, accompanied by atresia of external acoustic meatus (Figure 3); and type IV: anotia with atresia of the external acoustic meatus.

Among the 81 fetuses with microtia diagnosed after birth or induced labor, 2 cases were missed diagnosis on prenatal ultrasound, and 1 case was diagnosed as unilateral microtia by prenatal ultrasound but was found to be bilateral microtia after birth. The concordance rate of prenatal ultrasound diagnosis was 96.3%. Bilateral microtia was found in 2 cases and unilateral microtia in 79 cases, including 1 case of grade I, 17 cases of grade II, 62 cases of grade III and 3 cases of grade IV. Microtia was accompanied by an accessory auricle in 4 cases and low-set ears in 7 cases.

Twenty-two cases (27.16%) were complicated with other structural anomalies, including 11 cases (13.58%) of cardiac anomalies, 7 cases (8.64%) of ultrasonographic soft marker anomalies, 6 cases (7.41%) of facial anomalies, 6 cases (7.41%) of nervous system anomalies, 3 cases (3.70%) of urogenital system anomalies, 3 cases (3.70%) of digestive tract anomalies, and 2 cases (2.47%) of limb anomalies, as shown in Table 2.

**Table 2:** Prenatal ultrasound features, associated anomalies and genetic abnormalities of 81 fetuses with congenital microtia.

Associated abnormality	Positive signals
Other auricle abnormality	Low-set ears
	Accessory auricle
Nervous system anomalies	Sacrococcygeal teratoma
	Vertebral anomalies
	Corpus callosum dysgenesis, Dandy-Walker
	Corpus callosum dysgenesis
Facial anomalies	Micrognathia
	Micrognathia, cleft palate
	Unilateral transverse facial cleft
	Cleft palate
Thoracic cavity anomalies	Absent thymus, pulmonary dysplasia
	Diaphragmatic hernia, congenital cystadenoma malformation
Cardiac anomalies	Double outlet of right ventricle, Ventricular septal defect
	Pericardial effusion
	Ventricular septal defect
	Ventricular septal defect, persistent left superior vena cava
	Ventricular septal defect, aortic translocation
	Coarctation of aorta
Digestive tract anomalies	Esophageal atresia
	Duodenal atresia
Urogenital system anomalies	Hydronephrosis
	Ectopic kidney
	Horseshoe kidney
Limb anomalies	Hand posture abnormalities
	Overlapping fingers
	Knee posture abnormalities
Ultrasonographic soft marker anomalies	Oedema
	Hypoplastic placenta
	Nasal bone absence
	Polyhydramnios
	Cystic hygroma of the neck
	Abnormal septa pellucida
	Single umbilical artery
	Choroid plexus cyst, nasal bone absence
	Choroid plexus cyst, polyhydramnios
	Single umbilical artery

Chromosome karyotype analysis and gene detection were performed in 44 cases. Trisomy 18, trisomy 13, trisomy 21, pericentric inversion of chromosome 9, partial loss of heterozygosity on chromosome 14, 22q11 microdeletion and a normal karyotype were found in 2 cases, 2 cases, 3 cases, 1 case, 1 case, 1 case, and 34 cases, respectively.

## 4. Discussion

The external ear consists of the auricle and the external acoustic meatus. As important auditory organs, the auricle and the external acoustic meatus are mainly responsible for sound collection and aesthetics. The auricle is formed in the first branchial groove and the adjacent first and second branchial arches at the 5th–6th weeks of embryo development (3, 4). At the 20th week of embryo development, the anatomical morphology of the auricle is similar to that of adults. Blockage of this developmental process in the embryo can result in microtia characterized by a range of developmental anomalies of the auricle, including partial structural defects of the auricle and complete structural defects of the external ear, which are often accompanied by atresia of the external acoustic meatus, low-set ears and middle ear deformity along with varying degrees of conductive hearing impairment (5).

The incidence of congenital microtia in fetuses is 1‱–3‱, and the number of unilateral microtia cases is 3–5 times that of bilateral microtia cases, with unilateral microtia being more common in the right ear and in males. The incidence of microtia is positively correlated with maternal age, with an older age corresponding to a higher incidence. Due to ethnic differences, the Asian population is more susceptible to microtia (6–9).

2D ultrasound is the preferred method for fetal auricle observation. As reported in the literature, the second trimester is the best time to observe the fetal ear morphology (10). In this study, the median gestational age at diagnosis was 24^+1^ weeks, with a display rate of 97.53%, which is consistent with the conclusion in the above literature. Ultrasonically, the normal fetal auricle shows a clear and bright “C”- or “S”-type medium echo (4). The left and right auricles are symmetrical and almost equal in size. The normal auricle length measured on the parasagittal plane is linearly correlated with gestational age. The main prenatal ultrasound features of microtia include an obviously smaller length diameter of the auricle on the affected side than that on the unaffected side, disappearance of the normal auricle morphology on the affected side, which is replaced by punctate, lumpy or abnormal soft tissue echoes, and obvious asymmetry in the size and morphology of the bilateral fetal auricles on the horizontal transverse plane and coronal plane. In addition to the length of the auricles, the morphology and position of the auricles should also be observed on fetal auricle examination. In cases of fetal auricle abnormalities, the contralateral auricle should be examined carefully, and whether it is accompanied by other developmental abnormalities should be assessed. In this study, 1 case was diagnosed with unilateral microtia by prenatal ultrasound but with bilateral microtia of different degrees after birth and induced labor, and mild deformity occurred on the side with the missed diagnosis. Therefore, using the contralateral auricle as a reference for diagnosis is not recommended. Additionally, 2 cases of missed diagnosis on prenatal ultrasound were noted, but they were diagnosed after birth. According to the retrospective analysis of the images, only the unilateral auricle was shown in 1 case in the first ultrasound examination due to fetal position limitation, and the pregnant woman was instructed to undergo the examination again after a half-hour. However, the ipsilateral auricle was again mistaken for the contralateral auricle in the image. In another case, an image of the auricle was not obtained. 3D images have advantages in stereoscopic visual display of auricle deformity. However, due to the limitation of acquisition conditions including fetal position, amniotic fluid volume and other factors, 3D images acquisition was only performed in some cases.

Microtia is often accompanied by other systemic deformities. As described by Ye et al. (11) in a study involving 672 microtia patients, 1 or multiple associated abnormalities were found in 293 patients, including ear-face-neck abnormalities (40% of all associated abnormalities) and musculoskeletal system and cardiovascular system abnormalities. Research also suggests that microtia is often associated with renal dysplasia (12), with poorer auricle development corresponding to a higher risk of associated anomalies (13). Guo et al. (14) conducted a study on microtia in fetuses after birth and found that the prevalence of congenital heart disease among microtia patients is higher than that among the general population. Among the associated anomalies in this study, cardiac anomalies had the highest incidence, followed by ultrasonographic soft marker anomalies, facial anomalies and nervous system anomalies. The incidence of fetal microtia with cardiac anomalies remains high.

The pathogenesis of congenital microtia is still unclear but may involve multiple genetic and environmental factors. Microtia can be caused by many environmental risk factors, such as maternal anemia during pregnancy, diabetes mellitus, elderly age, multiple pregnancy and race. Some studies have also shown that tretinoin, thalidomide and mycophenolate mofetil are closely associated with microtia (15, 16).

Fetal auricle examination is not covered in prenatal ultrasound screening, but many recent studies have verified that microtia may indicate chromosomal abnormalities, especially triploidy (1). Chromosomal variations are considered to be associated with the occurrence of congenital microtia, especially in cases with multiple deformities or syndromic microtia. However, chromosomal variations have a lower incidence in nonsyndromic, isolated microtia (8).

Mortier et al. (7) performed gene detection on 44 microtia patients using single nucleotide polymorphism microarray technology. They found no pathological copy number variations that can explain the phenotype by genome-wide deletion repeat analysis using the microarray and argued that grade III microtia is the most common. Si et al. (17) found 2 cases of microtia associated with 22q11 deletion syndrome. In this study, one case of 22q11 microdeletion was found by genetic testing.

In this study, the pregnant women and their families had a less positive attitude toward genetic testing, and fetal chromosome karyotype analysis and gene detection were conducted on only a few cases; therefore, statistical analysis was performed. In addition, some studies suggest that fetal auricle abnormalities may be a new clinical indicator for intrauterine growth restriction, but more studies are required (18).

## 5. Conclusion

The results of this study demonstrated that prenatal ultrasound is reliable for diagnosing fetal microtia, and the second trimester is the best time to observe the fetal auricle. Unilateral fetal microtia is more common and often accompanied by congenital defects of other organs and structures, with frequent involvement of the heart and face. Soft marker anomalies are also of clinical significance.

## Data availability statement

The original contributions presented in the study are included in the article/Supplementary material, further inquiries can be directed to the corresponding author.

## Ethics statement

The studies involving human participants were reviewed and approved by Ethics committee of Tianjin Medical University Ethics committee of Tianjin First Central Hospital Ethics committee of Shandong Provincial Maternal and Child Health Care Hospital. The patients/participants provided their written informed consent to participate in this study.

## Author contributions

JQ: conceptualization, writing–original draft, data collection and processing, and literature search. YR: conceptualization, writing–original draft, genetic analysis, and discussion. YG: data collection. JS: supervision, review, and editing. All authors contributed to the article and approved the submitted version.

## Funding

This work was supported by the Science and Technology Innovation Research Special Fund Project of Shandong Provincial Maternal and Child Health Association.

## Conflict of interest

The authors declare that the research was conducted in the absence of any commercial or financial relationships that could be construed as a potential conflict of interest.

## Publisher’s note

All claims expressed in this article are solely those of the authors and do not necessarily represent those of their affiliated organizations, or those of the publisher, the editors and the reviewers. Any product that may be evaluated in this article, or claim that may be made by its manufacturer, is not guaranteed or endorsed by the publisher.
