# Supplementary material for: Experience in prenatal ultrasound diagnosis of fetal microtia and associated abnormalities
Source: Front Med (Lausanne). 2023 Jul 11;10:1119191. doi: 10.3389/fmed.2023.1119191 (PMC10367006; doi:10.3389/fmed.2023.1119191)
Supplement: Supplementary file 1 [file Table_1.docx]

# Experience in Prenatal Ultrasound Diagnosis of Fetal Microtia and Associated Abnormalities

**Table S1 Relevant details of 81 fetuses**

| Case # | Gestation Age at Diagnosis (weeks) | Type | Gender | unilateral/bilateral microtia | genetic features |
| --- | --- | --- | --- | --- | --- |
| 1 | 15 | II | M | bilateral | trisomy 18 |
| 2 | 23 | III | F | bilateral | trisomy 21 |
| 3 | 23 | II | M | unilateral | normal |
| 4 | 24 | IV | F | unilateral | trisomy 21 |
| 5 | 22 | III | M | unilateral | normal |
| 6 | 25 | III | F | unilateral | no relevant tests |
| 7 | 24 | II | M | unilateral | normal |
| 8 | 24 | III | F | unilateral | antenatal noninvasive DNA testing: normal, deafness gene:normal |
| 9 | 24 | III | F | unilateral | trisomy 13 |
| 10 | 24 | II | F | unilateral | normal |
| 11 | 24 | III | M | unilateral | normal |
| 12 | 24 | II | M | bilateral | normal |
| 13 | 24 | III | F | unilateral | antenatal noninvasive DNA testing: normal |
| 14 | 24 | I | M | unilateral | normal |
| 15 | 24 | III | F | unilateral | no relevant tests |
| 16 | 23 | II | M | unilateral | normal |
| 17 | 24 | III | F | unilateral | normal |
| 18 | 24 | III | F | unilateral | normal |
| 19 | 25 | III | M | unilateral | normal |
| 20 | 25 | III | M | unilateral | antenatal noninvasive DNA testing: normal |
| 21 | 25 | III | M | bilateral | no relevant tests |
| 22 | 26 | IV | M | bilateral | no relevant tests |
| 23 | 26 | III | M | unilateral | normal |
| 24 | 26 | III | M | unilateral | no relevant tests |
| 25 | 26 | III | F | unilateral | no relevant tests |
| 26 | 27 | II | M | unilateral | normal |
| 27 | 29 | III | M | unilateral | antenatal noninvasive DNA testing: normal |
| 28 | 24 | right-IIleft-III | F | bilateral | normal |
| 29 | 32/37 | III | F | unilateral | normal |
| 30 | 22 | II | M | unilateral | normal |
| 31 | 13 | III | F | bilateral | normal |
| 32 | 22 | III | F | unilateral | trisomy 18 |
| 33 | 22 | III | M | unilateral | 22q11 microdeletion and a normal karyotype |
| 34 | 23 | III | M | unilateral | no relevant tests |
| 35 | 23 | III | F | bilateral | no relevant tests |
| 36 | 23 | II | F | unilateral | normal |
| 37 | 23 | III | F | unilateral | no relevant tests |
| 38 | 23 | II | F | unilateral | normal |
| 39 | 23 | III | M | unilateral | no relevant tests |
| 40 | 23 | III | F | unilateral | partial loss of heterozygosity on chromosome 14 |
| 41 | 24 | III | F | bilateral | no relevant tests |
| 42 | 24 | III | F | unilateral | no relevant tests |
| 43 | 24 | III | M | unilateral | normal |
| 44 | 24 | III | M | unilateral | normal |
| 45 | 24 | III | F | unilateral | no relevant tests |
| 46 | 24 | III | M | unilateral | no relevant tests |
| 47 | 24 | III | M | unilateral | no relevant tests |
| 48 | 24 | III | F | unilateral | normal |
| 49 | 24 | III | M | unilateral | normal |
| 50 | 24 | III | M | unilateral | no relevant tests |
| 51 | 24 | III | F | unilateral | no relevant tests |
| 52 | 24 | III | M | unilateral | antenatal noninvasive DNA testing: normal |
| 53 | 24 | III | F | unilateral | no relevant tests |
| 54 | 24 | III | M | unilateral | pericentric inversion of chromosome 9 |
| 55 | 24 | III | M | unilateral | no relevant tests |
| 56 | 24 | III | M | unilateral | no relevant tests |
| 57 | 24 | III | F | unilateral | normal |
| 58 | 24 | III | M | unilateral | no relevant tests |
| 59 | 25 | III | M | unilateral | no relevant tests |
| 60 | 25 | III | F | bilateral | normal |
| 61 | 25 | III | M | unilateral | no relevant tests |
| 62 | 25 | III | F | unilateral | no relevant tests |
| 63 | 25 | III | M | unilateral | no relevant tests |
| 64 | 25 | II | F | unilateral | normal |
| 65 | 24 | III | M | bilateral | no relevant tests |
| 66 | 25 | III | M | unilateral | trisomy 13 |
| 67 | 25 | III | F | unilateral | no relevant tests |
| 68 | 25 | III | F | unilateral | normal |
| 69 | 26 | III | F | unilateral | no relevant tests |
| 70 | 26 | III | M | bilateral | no relevant tests |
| 71 | 26 | III | M | unilateral | no relevant tests |
| 72 | 26 | IV | M | unilateral | no relevant tests |
| 73 | 26 | III | M | unilateral | no relevant tests |
| 74 | 27 | II | F | unilateral | normal |
| 75 | 27 | II | M | unilateral | normal |
| 76 | 27 | II | F | unilateral | normal |
| 77 | 28 | II | F | unilateral | normal |
| 78 | 25 | left-II right-III | F | bilateral | no relevant tests |
| 79 | 26 | III | F | unilateral | trisomy 21 |
| 80 | missed diagnosis | / | M | / | normal |
| 81 | missed diagnosis | III | M | unilateral | normal |
